# Modeling the Potential Impact of Host Population Survival on the Evolution of *M. tuberculosis* Latency

**DOI:** 10.1371/journal.pone.0105721

**Published:** 2014-08-26

**Authors:** Nibiao Zheng, Christopher C. Whalen, Andreas Handel

**Affiliations:** 1 Institute of Bioinformatics, University of Georgia, Athens, Georgia, United States of America; 2 Department of Epidemiology and Biostatistics, College of Public Health, University of Georgia, Athens, Georgia, United States of America; Swiss Tropical and Public Health Institute, Switzerland

## Abstract

Tuberculosis (TB) is an infectious disease with a peculiar feature: Upon infection with the causative agent, *Mycobacterium Tuberculosis* (MTB), most hosts enter a latent state during which no transmission of MTB to new hosts occurs. Only a fraction of latently infected hosts develop TB disease and can potentially infect new hosts. At first glance, this seems like a waste of transmission potential and therefore an evolutionary suboptimal strategy for MTB. It might be that the human immune response keeps MTB in check in most hosts, thereby preventing it from achieving its evolutionary optimum. Another possible explanation is that long latency and progression to disease in only a fraction of hosts are evolutionary beneficial to MTB by allowing it to persist better in small host populations. Given that MTB has co-evolved with human hosts for millenia or longer, it likely encountered small host populations for a large share of its evolutionary history and had to evolve strategies of persistence. Here, we use a mathematical model to show that indeed, MTB persistence is optimal for an intermediate duration of latency and level of activation. The predicted optimal level of activation is above the observed value, suggesting that human co-evolution has lead to host immunity, which keeps MTB below its evolutionary optimum.

## Introduction

Tuberculosis (TB) is an infectious disease caused by the bacterium *Mycobacterium tuberculosis* (MTB). MTB has been infecting humans for a long time, at least millenia and likely even longer [1]. As such, MTB had to evolve evolutionary strategies that allowed it to persist in small groups of human hosts. An interesting question is if one can see signatures of this potential evolutionary adaptation to small groups of human hosts in MTB's “life history” today.

Upon infection with MTB, most hosts enter a latent state. Those hosts do not show signs of disease but do harbor MTB, which can activate and lead to disease at any future time [2]–[4]. It might take a long time before activation occurs, and the majority of TB infected hosts die from other causes besides TB without ever developing disease [5]. Hosts latently infected with MTB cannot infect others. Therefore, at first glance, latency does not seem to be beneficial for MTB. One possible explanation for the long latency and the fact that activation to disease only occurs in a fraction of hosts is based on the human immune response [6]–[8]. It is known that a competent immune response is needed to contain infection and avoid disease, as dramatically illustrated in HIV infected hosts with weakened immune responses, who activate at much higher rates [9]–[11]. This suggests that the co-evolutionary arms race between MTB and its human hosts has lead to a host immune response that can successfully contain MTB in a state of suboptimal fitness [12], [13]. The fact that there seems to be local adaptation on both the pathogen and host side lends support to this idea [13]–[17].

Although host immune pressure is a plausible explanation for reduced activation rates, there are also arguments against this idea. Pathogens like MTB replicate rapidly (compared to their human hosts) and often reach large population sizes; both features are known to foster rapid evolution, especially under strong selective pressure [18], [19]. This is at vivid display in the evolution of drug resistance for MTB and many other pathogens [20], [21]. Therefore, one could argue that if long latency and low activation rates were evolutionary strongly suboptimal for MTB, evolution would have led to higher rates of activation. Instead, as has been suggested previously [22], [23], long latency and low activation rates might be strategies that are evolutionary beneficial to MTB by increasing its fitness.

There is no single way to quantify the fitness of an organism. For infectious diseases, an often used measure of fitness is transmissibility, usually defined by the reproductive number, *R*_0_ [24], [25]. It can be shown that in direct competition of two strains (under equilibrium conditions), the strain with the higher *R*_0_ outcompetes the one with the lower *R*_0_ [25]. However, in the absence of direct competition, strains with lower *R*_0_ might sometimes be more advantaged as they can better avoid local extinction and therefore win through indirect competition against strains with higher transmissibility [26]–[29]. Therefore, an alternative way to capture fitness of pathogens is by quantifying their ability to persist in a host population.

The impact of transmissibility and persistence on overall fitness has been an active area of research [30]–[34]. The importance of different measures of fitness such as these depends on the situation. Since MTB is an ancient organism that has infected humans for millenia [12], [35]–[38], it had to evolve strategies that allowed it to persist in relatively small, spatially structured host populations. Under such circumstances, fitness benefits due to non-extinction might have had an important impact on overall evolutionary dynamics [28], [39], [40].

It has been previously proposed that a prolonged latency might have been one persistence strategy [23], [41]. Here, we use a mathematical model to explore this idea. We introduce a measure of persistence of MTB in a population and investigate how well MTB can persist as a function of the latent period. We find that TB persistence is optimal for an intermediate duration of latency and level of activation. We also find that the optimal level of activation is above the observed value, suggesting that host immunity plays some role in keeping MTB below its optimal level of fitness.

## Methods

### Mathematical model description

**Figure 1:**
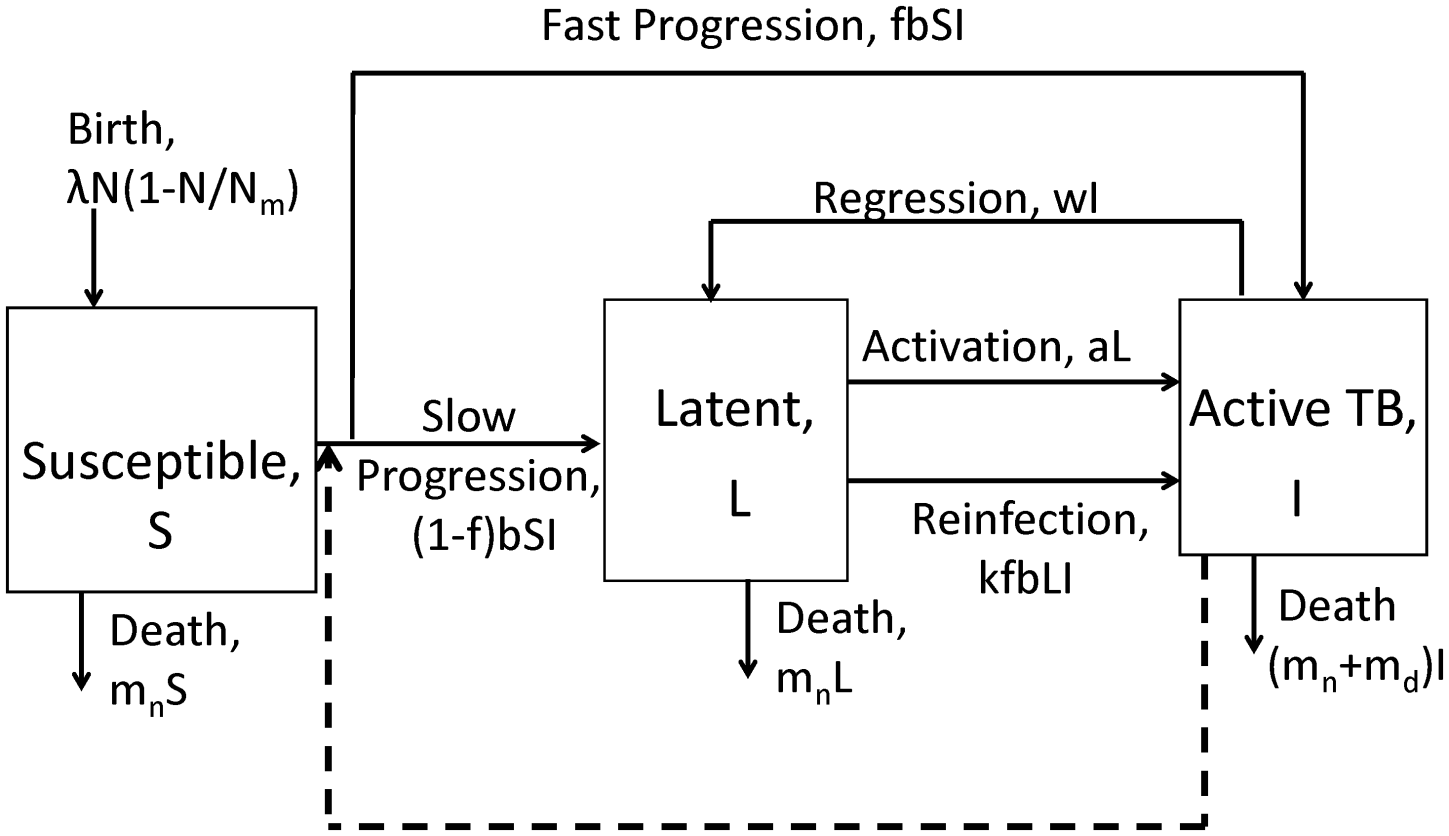
Flow diagram for the TB transmission model. Susceptible hosts (S) are born at rate 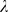 with birth rate saturating at high population density, *N_m_*. Hosts in each compartment die at a background mortality rate *m_n_*. Infection of susceptibles occurs at rate *b* upon contact with TB infectious hosts (I). After TB infection, a fraction *f* of hosts develop active TB in a short time (fast progression), the remainder enter the latent stage. Latent hosts (L) can activate and develop TB disease sometime later at rate *a* (slow progression). Latent hosts can also develop active TB after re-infection with TB, with probability of this occurring compared to fast progression of susceptibles reduced by a factor *k*. Infectious, diseased hosts (I) might regress to the latent stage at rate *w*. Hosts with active disease die due to diseased induced mortality at rate *m_d_*.

We use a compartmental mathematical model formulated as a set of ordinary differential equations to describe the population-level infection dynamics of TB. The model is shown schematically in figure 1. Our model is similar to other recently studied TB models [42], [43]. We consider 3 types of hosts (compartments) in our model: susceptible hosts, *S*, latently infected hosts, *L*, that harbor MTB but are not infectious and show no signs of disease, and infectious hosts with active disease, *I*. We keep the model simple and do not distinguish between features such as age-related differences (e.g. children versus adults). Such additional details could be included in further more detailed models. The total population size is *N* = *S*+*L*+*I*. New hosts enter the system at a maximum rate 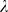 per person, this rate saturates once the population reaches some maximum level, *N_m_*. All hosts die due to causes other than TB after an average lifespan of 1/*m_n_* years. Uninfected hosts can become infected through contact with an infectious host at rate *b*. The infection process is modeled in a density dependent manner [43], [44]. After infection, a small fraction *f* of hosts rapidly develop disease (fast progression) [11], [45], [46] and move into the active disease compartment, *I*. The majority of hosts enter the latent state, *L* (slow progression). Latently infected hosts can convert to infectious, diseased hosts later in their life at rate *a* (activation) or through reinfection, with the chance of disease due to reinfection reduced by a factor *k* [47]–[49]. Infectious, diseased hosts either die due to disease after on average 1/*m_d_* years, or regress and return to the latent stage at rate *w*. Following previous models, we assume that recovered individuals do not fully clear the infection but instead return to the latent stage [43], [50]–[52]. Table 1 summarizes the model variables and parameters and provides references for the parameter values used for most of our analysis. The model equations are
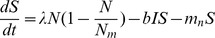
(1)

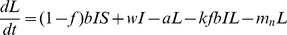
(2)

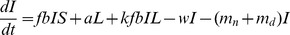
(3)


**Table 1:** Initial conditions and parameter values.

Symbol	Interpretation	Value	Source and Comments
*N_m_*	maximum population size	1	population size normalized to 1
*S*_0_	initial susceptible hosts	0.6	calculated as 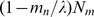 , to obtain steady population size in the absence of TB
*L*_0_	initial latent hosts	0	arbitrary choice
*I*_0_	initial infectious, diseased hosts	*S*_0_/1000	one infected per 1,000 hosts
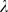	maximum birth rate	0.05 per year	50 births per 1,000 hosts, representing a high birth rate scenario
*m_n_*	natural mortality rate	0.02 per year	assuming a life-span of 50 years for healthy hosts
*m_d_*	disease-induced mortality rate	0.33 per year	assuming 3 years life-span for untreated diseased hosts [81]
*b*	rate of transmission	10 per year	[82]–[84]
*w*	rate of regression	0.25 per year	return from active TB stage to latency [81]
*f*	fraction of TB infections that lead to disease via fast progression	0.1	[11], [46], [50], [84]
*k*	reduction of fast progression rate upon reinfection of latent hosts	0.5	[45], [46], [49]
*a*	rate of TB activation in latent hosts	varied	

Initial conditions of model variables and values of model parameters. These values are chosen for all simulations unless indicated otherwise.

We implemented all model simulations in R [53]. The code to reproduce the simulations described here is available from the author's webpage at http://handelgroup.uga.edu/resources.htm.

## Results

### Persistence of MTB as a function of latency

Our main outcome of interest is the potential of MTB to persist in a host population and how persistence potential depends on the duration of the latent period and fraction of latent TB hosts that activate. While pathogens that have an environmental stage as an important component of their transmission cycle can persist even in the absence of any infected hosts [54]–[58], for pathogens where direct transmission is the main important component, persistence (non-extinction) requires the continued presence of infected hosts. For MTB, extinction occurs if no more latently infected hosts, *L*, and infectious, diseased hosts, *I*, are present. Persistence is more likely (i.e. extinction is less likely) as the number of infected hosts in the population increases [59]–[61]. Since for MTB, only a fraction of latently infected hosts will become infectious and contribute to transmission, a better measure of persistence is given by the quantity *P*, defined as 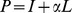, where *I* and *L* are the number of infectious and latently infected hosts, and 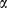 represents the fraction of latently infected hosts that will develop TB disease, become infectious and are able to transmit. For our model, 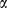 is given by the ratio of the rate of latently infected hosts that move on to develop active TB, 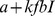, divided by the total rate at which latently infected hosts leave the latent stage, *a*+*kfbI+m_n_*, i.e. 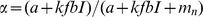.

**Figure 2:**
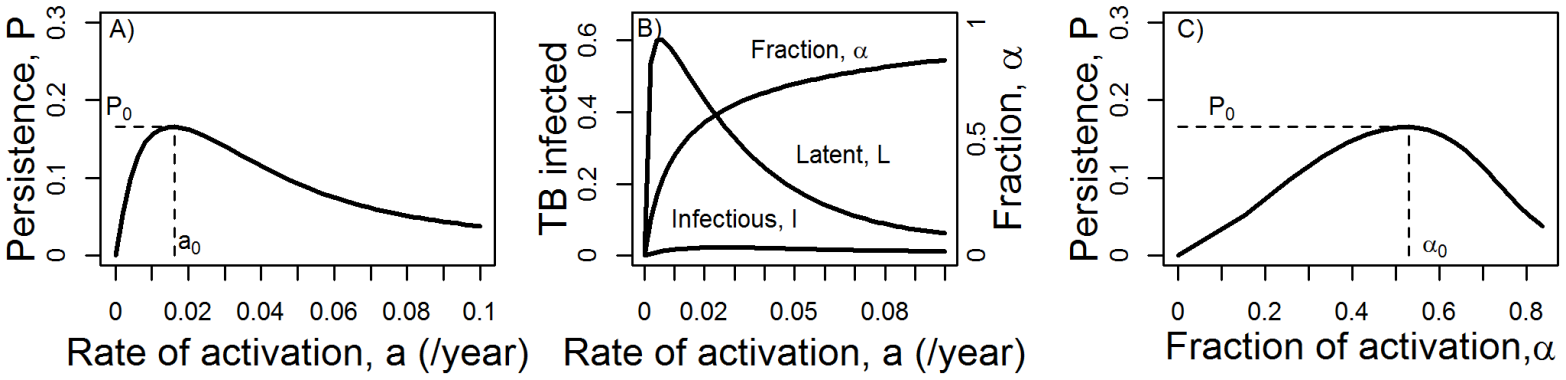
Persistence as function of activation. A) Persistence, *P*, as a function of activation rate. B) Latent and infectious hosts, 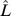 and 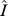, and the fraction of activation, 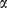, as a function of activation rate. The left axis applies to *L* and *I*, the right axis to 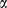. C) Persistence as function of fraction of hosts that activate. Also indicated in the figures is the optimal level of persistence, *P*_0_, and the values for activation rate and fraction of activators at which this optimum occurs, 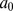 and 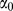. All parameters are as given in table 1.

Persistence as defined by *P*, especially at the steady state, provides a useful measure for the ability of MTB to not go extinct in the population (see our comparison with a stochastic model below). We run simulations of our model for different values of the activation rate, *a*, and record *I* and *L* at steady state and use this to compute *P* as function of *a*. Figure 2A shows that optimal persistence is achieved at intermediate rates of activation. Figure 2B shows individually the three components that make up *P*. The number of infectious hosts at steady state, as well as the fraction of hosts activating, 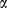, increase with increasing activation rate. The number of latent hosts first increases and then decreases with larger activation rate. The combination of these three quantities leads to a maximum for persistence *P* at intermediate values.

Optimal persistence at an intermediate rate of activation also implies that instead of having every latent host activate and become infectious, it is beneficial for the pathogen to let some infections “go to waste” by way of latent hosts dying before they become infectious. This helps with overall persistence and is worth the “loss” of a fraction of latent hosts due to natural death before they activate and are able to transmit. Figure 2C shows persistence as a function of the fraction of hosts that activate, 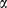. The figure also illustrates another interesting point: The optimal fraction of hosts that activate is slightly above 50% given the chosen model parameters. This is higher than the ≈10–20% observed [62], [63], suggesting that MTB is not able to achieve the activation rate that would optimize its persistence. This might be attributable to the host immune response playing a role at reducing activation.

### The impact of parameter value uncertainty on optimal persistence and activation

**Figure 3:**
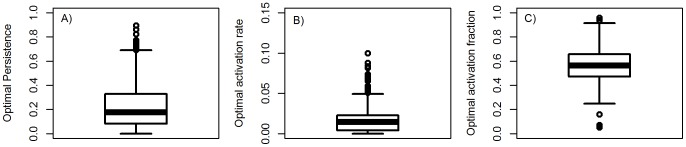
Uncertainty analysis of model results. Impact of model parameter variations on A) optimal persistence, *P*_0_, B) optimal rate of activation, *a*_0_, and C) optimal fraction of activation, 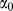. Model parameters were sampled in a range of 0.5–2 times the base parameters listed in table 1. Sampling was done using a latin hypercube approach with 1000 samples. Samples for which parameter combinations lead to a biologically unreasonable scenario, specifically natural mortality rate above birth rate, were discarded. For each sample, persistence as function of activation rate and activation fraction was determined (as shown in figure 2) and from this the optimal values for persistence and activation were obtained.

To investigate how sensitive results reported in the previous section are to changes in parameter values, we performed an uncertainty analysis and sampled the model parameters using Latin Hypercube Sampling [64]–[67]. For each parameter, we considered uniform distributions ranging between 0.5 and 2 times the base parameter value shown in table 1. For each parameter sample we computed the values *P*_0_, *a*_0_ and 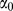, i.e. the optimal level of persistence and the activation rate and fraction at which the optimum occurs. Figure 3 shows the distribution of those values. Of most interest, for almost all parameter samples the fraction of hosts that activate remains well above the 10% found for MTB in the field.

In text S1, we show further results by exploring how changes in each model parameter individually affect optimal persistence and activation. As expected, increased population size (though increased carrying capacity parameter or increased birth rate) leads to improved persistence, while decreased population size (through higher death rates) reduces persistence. Other model parameters have less impact on changes in persistence. The optimal fraction of activators as any of these parameters are changed individually is between 40%–70%, again above the value observed experimentally.

We also explore in text S1 how changes in the model structure, specifically the assumption of exponentially distributed natural lifespans, affect our results. We find that it shifts the persistence curve shown in figure 2 slightly, without affecting the overall results and still giving an optimal fraction of activators around 50%.

### The deterministic persistence measure is well reproduced with a stochastic model

**Figure 4:**
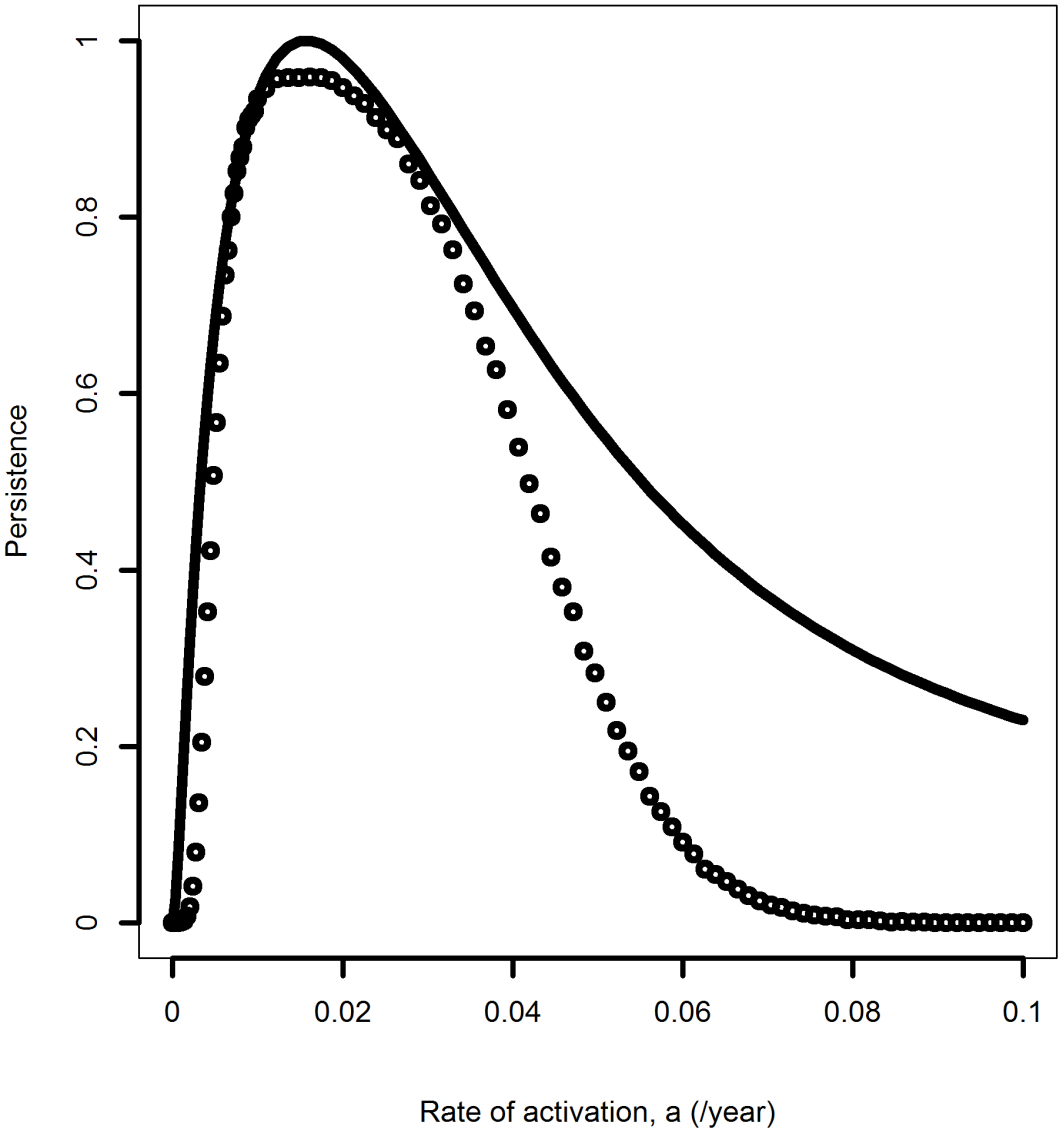
Comparison of persistence with results from a stochastic model. The solid line shows persistence, *P*, as determined from the deterministic model, symbols show result from a stochastic model. For the stochastic simulation, we started at the deterministic steady state, simulated the model 10,000 times for 1000 years each and counted the numbers of simulations for which at least one latent or infectious host was still present after 1000 years. A population of size 100 was used, all parameter values are as reported in table 1. Note that because the absolute magnitude of *P* is arbitrary and scales with population size, *N_m_*, to allow comparison with the stochastic model we rescaled *P* to be between 0 and 1.

Our persistence measure, *P*, is derived from a deterministic model. Of course, non-persistence, i.e. extinction, is an inherently stochastic process. While it is generally well known that larger population sizes lead to less chance of extinction [59]–[61], it is useful to directly test our deterministic measure with a stochastic model. To that end, we implemented the differential equations as a compartmental stochastic model, with transition rates of the deterministic model becoming transition probabilities [25], [68]. We simulated the stochastic model using an efficient form of the Gillespie algorithm (the adaptive-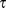 leap method as implemented in the R package adaptivetau [69], [70]). Starting at the deterministic equilibrium state, we simulated the stochastic model for a fixed number of years and record the fraction of simulations for which at least one infectious or latent individual was still present at the end of the simulation. For the stochastic model, there is no discounting of the latent hosts by a factor 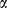. Instead, persistence or extinction is a binary event, with extinction defined as no more latent or diseased infected hosts present and persistence if at least one of these hosts was still present. Figure 4 shows that despite this difference between *P* and the stochastic simulation, the fraction of simulations for which persistence was found in the stochastic model has a very similar functional shape as our deterministic persistence measure, *P*, with the optimum, *P*_0_, occurring at more or less the same rate of activation.

In Text S1, we use the stochastic model to investigate how population growth or decline influence *P*. We find that persistence improves in the presence of a growing population and worsens if the population declines. The shape of the persistence curve does not change in any important way.

### Persistence during epidemic cycles

**Figure 5:**
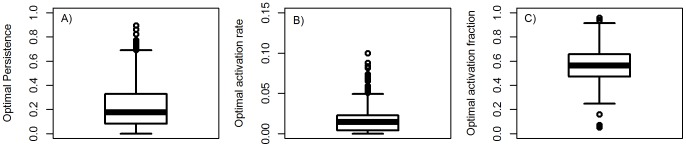
Optimal persistence, *P_m_*, activation rate and fraction for the non-steady state model. Everything as explained in caption for figure 3.

We have so far focused on MTB persistence at the endemic state. Equally important for pathogens is the ability to persist after introduction to a newly susceptible population. During TB's evolutionary history, it likely got introduced and re-introduced repeatedly into small susceptible subpopulations (e.g. new tribes/villages). In the evolutionary context, persistence upon introduction into a fully susceptible population might therefore have played an important role. Nowadays, many areas of the world where TB is very rare consist of largely susceptible individuals – though for those groups the persistence idea explored here is likely not too important. Upon entering a fully susceptible population, pathogens usually cause an epidemic outbreak, depleting the number of susceptibles. The outbreak is often followed by a fade-out of the disease once most susceptibles have been depleted. Extinction often occurs during this fade-out. For the pathogen to not go extinct, it needs to persist long enough until the number of susceptibles has built up again, usually leading to consecutive smaller outbreaks until the endemic state is reached [71], [72]. We can quantify persistence during epidemic cycles by evaluating our expression for persistence not at the steady state but instead at the overall minimum occurring between introduction of the disease in a susceptible population and eventual attainment of the endemic equilibrium, i.e. we determine the overall minimum 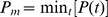. Figure 5 shows the distribution of optimal persistence, optimal activation rate and optimal fraction of activating hosts for *P_m_*, using the same parameter sampling approach as for figure 3. Comparing the results with those found earlier for *P* at the steady state (figure 3), one sees that the results are very similar. The main reason for the similarity is that TB has a relatively “slow” disease dynamics [73], without pronounced outbreak peaks and minima. Therefore, for most parameter values, the disease reaches the steady state without a large contraction after the first outbreak, leading to essentially the same results as for the steady state.

Again, as for *P* above, we show additional results for *P_m_* as function of individual parameters in Text S1. The results are almost identical to those obtained for *P*, for the reason just explained.

## Discussion

Recent in-depth studies of MTB genetic sequences have shown a wide diversity between strains [17]. While harder to determine, there is also accumulating evidence that this genetic diversity results in phenotypic diversity [17], [74], suggesting that MTB evolution is shaped by local selection pressures. A recent study indicates that disease activation rate differs between lineages, suggesting that this phenotype is under evolutionary selection [75]. We used a mathematical model to investigate the role of activation rate and latency duration on the ability of MTB to persist in a host population. Our results support the previously proposed idea that the prolonged latency observed for TB infections might provide MTB an evolutionary advantage, namely improved persistence in a host population [23], [41]. We found that an intermediate rate of disease activation is optimal for persistence.

Interestingly, our model suggests that for optimal persistence, the fraction of hosts that eventually become cases is in the 20%–80% range with approximately 50% as the most likely value. This range is higher than the ≈10% generally seen for TB, suggesting that the host immune response plays some role in keeping TB disease in check, lowering activation rate below what would be the evolutionary optimal level for the pathogen. It is likely that some level of co-evolution between pathogen and host occurred and that humans who have been exposed to MTB for a long time evolved some level of resistance that potentially prevents MTB from reaching its evolutionary optimal activation rate [12]. This also agrees with the observation that upon contact with novel MTB strains, some populations have been shown to experience much higher rates of disease than the usually observed 10% [76]. It is further interesting to note that the optimal rate of activation we find in our study is similar to values reported for HIV positive TB patients [77]–[79]. While the simplicity of our model is a caveat in interpreting this agreement too quantitatively, we believe our results provide another suggestive indication that the host immune response is responsible for keeping MTB activation rates below a value that would be optimal for MTB, and once the immune protection fails, as in HIV infected hosts, MTB activates at rates close to its optimum value.

In summary, our results suggest that an intermediate level of activation from latency to disease is optimal for MTB persistence, that the optimal level depends on the detailed pathogen, host and environment characteristics, and that it tends to be higher than the observed value, suggesting an important role for the immune response to keep MTB in check. While increasing activation rates beyond the optimum to reduce MTB persistence is not a suitable goal from a public health perspective, a reduction in activation rate is much more promising. This would lower the number of hosts with disease, and thereby reduce incidence and prevalence for TB cases and at the same time reduce persistence potential. Potential TB vaccines currently under consideration might help us to achieve such a reduction in activation rate [80].

## Supporting Information

Text S1 Additional results and explanations.
(PDF)

## References

[1] Brites D, Gagneux S (2012) Old and new selective pressures on mycobacterium tuberculosis. Infect Genet Evol 12: 678–685. 21867778 10.1016/j.meegid.2011.08.010 PMC3253320

[2] Gomez JE, McKinney JD (2004) M. tuberculosis persistence, latency, and drug tolerance. Tuberculosis (Edinb) 84: 29–44. 14670344 10.1016/j.tube.2003.08.003

[3] Barry CE 3rd, Boshoff HI, Dartois V, Dick T, Ehrt S, et al (2009) The spectrum of latent tuberculosis: rethinking the biology and intervention strategies. Nat Rev Microbiol 7: 845–855. 19855401 10.1038/nrmicro2236 PMC4144869

[4] Kirschner DE, Young D, Flynn JL (2010) Tuberculosis: global approaches to a global disease. Curr Opin Biotechnol 21: 524–531. 20637596 10.1016/j.copbio.2010.06.002 PMC2943033

[5] Frieden TR, Sterling TR, Munsiff SS, Watt CJ, Dye C (2003) Tuberculosis. Lancet 362: 887–899. 13678977 10.1016/S0140-6736(03)14333-4

[6] Chan J, Flynn J (2004) The immunological aspects of latency in tuberculosis. Clin Immunol 110: 2–12. 14986673 10.1016/s1521-6616(03)00210-9

[7] Young D, Stark J, Kirschner D (2008) Systems biology of persistent infection: Tuberculosis as a case study. Nat Rev Microbiol 6: 520–528. 18536727 10.1038/nrmicro1919

[8] Cooper AM (2009) Cell-mediated immune responses in tuberculosis. Annu Rev Immunol 27: 393–422. 19302046 10.1146/annurev.immunol.021908.132703 PMC4298253

[9] Bauer AL, Hogue IB, Marino S, Kirschner DE (2008) The effects of hiv-1 infection on latent tuberculosis. Mathematical Modelling of Natural Phenomena 3: 229–266.

[10] Corbett EL, Watt CJ, Walker N, Maher D, Williams BG, et al (2003) The growing burden of tuberculosis: global trends and interactions with the hiv epidemic. Arch Intern Med 163: 1009–21. 12742798 10.1001/archinte.163.9.1009

[11] Whalen CC, Zalwango S, Chiunda A, Malone L, Eisenach K, et al (2011) Secondary attack rate of tuberculosis in urban households in kampala, uganda. PLoS One 6: e16137. 21339819 10.1371/journal.pone.0016137 PMC3038854

[12] Gagneux S (2012) Host-pathogen coevolution in human tuberculosis. Philos Trans R Soc Lond B Biol Sci 367: 850–859. 22312052 10.1098/rstb.2011.0316 PMC3267123

[13] Comas I, Gagneux S (2011) A role for systems epidemiology in tuberculosis research. Trends Microbiol 19: 492–500. 21831640 10.1016/j.tim.2011.07.002 PMC3184389

[14] Gagneux S, Small PM (2007) Global phylogeography of mycobacterium tuberculosis and implications for tuberculosis product development. Lancet Infect Dis 7: 328–337. 17448936 10.1016/S1473-3099(07)70108-1

[15] Caws M, Thwaites G, Dunstan S, Hawn TR, Lan NTN, et al (2008) The influence of host and bacterial genotype on the development of disseminated disease with mycobacterium tuberculosis. PLoS Pathog 4: e1000034. 18369480 10.1371/journal.ppat.1000034 PMC2268004

[16] Mller M, de Wit E, Hoal EG (2010) Past, present and future directions in human genetic susceptibility to tuberculosis. FEMS Immunol Med Microbiol 58: 3–26. 19780822 10.1111/j.1574-695X.2009.00600.x

[17] Coscolla M, Gagneux S (2010) Does m. tuberculosis genomic diversity explain disease diversity? Drug Discov Today Dis Mech 7: e43–e59. 21076640 10.1016/j.ddmec.2010.09.004 PMC2976975

[18] Holmes EC (2009) The Evolutionary Genetics of Emerging Viruses. ANNUAL REVIEW OF ECOLOGY EVOLUTION AND SYSTEMATICS 40: 353–372.

[19] Kawecki TJ, Lenski RE, Ebert D, Hollis B, Olivieri I, et al (2012) Experimental evolution. Trends Ecol Evol 27: 547–560. 22819306 10.1016/j.tree.2012.06.001

[20] Levy SB, Marshall B (2004) Antibacterial resistance worldwide: causes, challenges and responses. Nature Medicine 10: S122–S129. 10.1038/nm1145 15577930

[21] Gandhi NR, Nunn P, Dheda K, Schaaf HS, Zignol M, et al (2010) Multidrug-resistant and extensively drug-resistant tuberculosis: a threat to global control of tuberculosis. Lancet 375: 1830–1843. 20488523 10.1016/S0140-6736(10)60410-2

[22] Flynn JL, Chan J (2005) What's good for the host is good for the bug. Trends Microbiol 13: 98–102. 15737727 10.1016/j.tim.2005.01.005

[23] Blaser MJ, Kirschner D (2007) The equilibria that allow bacterial persistence in human hosts. Nature 449: 843–849. 17943121 10.1038/nature06198

[24] Heffernan JM, Smith RJ, Wahl LM (2005) Perspectives on the basic reproductive ratio. J R Soc Interface 2: 281–293. 16849186 10.1098/rsif.2005.0042 PMC1578275

[25] Keeling M, Rohani P (2007) Modeling Infectious Diseases in Humans and Animals. Princeton University Press.

[26] Rankin DJ, Bargum K, Kokko H (2007) The tragedy of the commons in evolutionary biology. Trends Ecol Evol 22: 643–651. 17981363 10.1016/j.tree.2007.07.009

[27] Lion S, Baalen Mv (2008) Self-structuring in spatial evolutionary ecology. Ecol Lett 11: 277–295. 18070102 10.1111/j.1461-0248.2007.01132.x

[28] Messinger SM, Ostling A (2009) The consequences of spatial structure for the evolution of pathogen transmission rate and virulence. Am Nat 174: 441–454. 19691436 10.1086/605375

[29] Parvinen K, Dieckmann U (2013) Self-extinction through optimizing selection. J Theor Biol 333: 1–9. 23583808 10.1016/j.jtbi.2013.03.025 PMC3730061

[30] Lenski RE, May RM (1994) The evolution of virulence in parasites and pathogens: reconciliation between two competing hypotheses. J Theor Biol 169: 253–265. 7967617 10.1006/jtbi.1994.1146

[31] Keeling M (2000) Evolutionary trade-offs at two time-scales: competition versus persistence. Proc Biol Sci 267: 385–391. 10722221 10.1098/rspb.2000.1013 PMC1690535

[32] Day T, Gandon S (2007) Applying population-genetic models in theoretical evolutionary epidemiology. Ecol Lett 10: 876–888. 17845288 10.1111/j.1461-0248.2007.01091.x

[33] Bahi-Jaber N, Fouchet D, Pontier D (2008) Stochastic extinction and the selection of the transmission mode in microparasites. J R Soc Interface 5: 1031–1039. 18238759 10.1098/rsif.2007.1326 PMC2607425

[34] King AA, Shrestha S, Harvill ET, Bjrnstad ON (2009) Evolution of acute infections and the invasion-persistence trade-off. Am Nat 173: 446–455. 19231966 10.1086/597217 PMC4101379

[35] Donoghue HD, Spigelman M, Greenblatt CL, Lev-Maor G, Bar-Gal GK, et al (2004) Tuberculosis: from prehistory to robert koch, as revealed by ancient dna. Lancet Infect Dis 4: 584–592. 15336226 10.1016/S1473-3099(04)01133-8

[36] Wilbur AK, Farnbach AW, Knudson KJ, Buikstra JE (2008) Diet, tuberculosis, and the paleopathological record. Curr Anthropol 49: 963–77 discussion 977–91. 19391442 10.1086/592434

[37] Hershberg R, Lipatov M, Small PM, Sheffer H, Niemann S, et al (2008) High functional diversity in mycobacterium tuberculosis driven by genetic drift and human demography. PLoS Biol 6: e311. 19090620 10.1371/journal.pbio.0060311 PMC2602723

[38] Smith NH, Hewinson RG, Kremer K, Brosch R, Gordon SV (2009) Myths and misconceptions: the origin and evolution of mycobacterium tuberculosis. Nat Rev Microbiol 7: 537–544. 19483712 10.1038/nrmicro2165

[39] Haraguchi Y, Sasaki A (2000) The evolution of parasite virulence and transmission rate in a spatially structured population. J Theor Biol 203: 85–96. 10704294 10.1006/jtbi.1999.1065

[40] Kerr B, Neuhauser C, Bohannan BJM, Dean AM (2006) Local migration promotes competitive restraint in a host-pathogen ‘tragedy of the commons’. Nature 442: 75–78. 16823452 10.1038/nature04864

[41] Ernst JD, Trevejo-Nuez G, Banaiee N (2007) Genomics and the evolution, pathogenesis, and diagnosis of tuberculosis. J Clin Invest 117: 1738–1745. 17607348 10.1172/JCI31810 PMC1904327

[42] Castillo-Chavez C, Song B (2004) Dynamical models of tuberculosis and their applications. Math Biosci Eng 1: 361–404. 20369977 10.3934/mbe.2004.1.361

[43] Basu S, Galvani AP (2009) The evolution of tuberculosis virulence. Bull Math Biol 71: 1073–1088. 19172358 10.1007/s11538-009-9394-x

[44] Begon M, Bennett M, Bowers RG, French NP, Hazel SM, et al (2002) A clarification of transmission terms in host-microparasite models: numbers, densities and areas. Epidemiol Infect 129: 147–153. 12211582 10.1017/s0950268802007148 PMC2869860

[45] Sutherland I, Svandova E, Radhakrishna S (1982) The development of clinical tuberculosis following infection with tubercle bacilli. 1. a theoretical model for the development of clinical tuberculosis following infection, linking from data on the risk of tuberculous infection and the incidence of clinical tuberculosis in the netherlands. Tubercle 63: 255–68. 6763793 10.1016/s0041-3879(82)80013-5

[46] Vynnycky E, Fine PE (1997) The natural history of tuberculosis: the implications of age-dependent risks of disease and the role of reinfection. Epidemiol Infect 119: 183–201. 9363017 10.1017/s0950268897007917 PMC2808840

[47] Chiang CY, Riley LW (2005) Exogenous reinfection in tuberculosis. Lancet Infect Dis 5: 629–636. 16183517 10.1016/S1473-3099(05)70240-1

[48] Cohen T, Colijn C, Finklea B, Murray M (2007) Exogenous re-infection and the dynamics of tuberculosis epidemics: local effects in a network model of transmission. Journal of The Royal Society Interface 4: 523–531. 10.1098/rsif.2006.0193 PMC2373405 17251134

[49] Andrews JR, Noubary F, Walensky RP, Cerda R, Losina E, et al (2012) Risk of progression to active tuberculosis following reinfection with mycobacterium tuberculosis. Clin Infect Dis 54: 784–791. 22267721 10.1093/cid/cir951 PMC3284215

[50] Bacaer N, Ouifki R, Pretorius C, Wood R, Williams B (2008) Modeling the joint epidemics of tb and hiv in a south african township. J Math Biol 57: 557–93. 18414866 10.1007/s00285-008-0177-z

[51] Williams BG, Granich R, Chauhan LS, Dharmshaktu NS, Dye C (2005) The impact of hiv/aids on the control of tuberculosis in india. Proc Natl Acad Sci U S A 102: 9619–24. 15976029 10.1073/pnas.0501615102 PMC1157104

[52] Guzzetta G, Ajelli M, Yang Z, Merler S, Furlanello C, et al (2011) Modeling socio-demography to capture tuberculosis transmission dynamics in a low burden setting. J Theor Biol 289: 197–205. 21906603 10.1016/j.jtbi.2011.08.032 PMC3208139

[53] R Development Core Team (2013). R: A language and environment for statistical computing. http://www.r-project.org/. ISBN 3-900051-07-0.

[54] Bonhoeffer S, Lenski RE, Ebert D (1996) The curse of the pharaoh: the evolution of virulence in pathogens with long living propagules. Proc Biol Sci 263: 715–721. 8763793 10.1098/rspb.1996.0107

[55] Walther BA, Ewald PW (2004) Pathogen survival in the external environment and the evolution of virulence. Biol Rev Camb Philos Soc 79: 849–869. 15682873 10.1017/S1464793104006475 PMC7161823

[56] Handel A, Bennett MR (2008) Surviving the bottleneck: transmission mutants and the evolution of microbial populations. Genetics 180: 2193–2200. 18854584 10.1534/genetics.108.093013 PMC2600951

[57] Caraco T, Wang IN (2008) Free-living pathogens: life-history constraints and strain competition. J Theor Biol 250: 569–579. 18062992 10.1016/j.jtbi.2007.10.029 PMC2262931

[58] Handel A, Brown J, Stallknecht D, Rohani P (2013) A multi-scale analysis of influenza a virus fitness trade-offs due to temperature-dependent virus persistence. PLoS computational biology 9: e1002989. 23555223 10.1371/journal.pcbi.1002989 PMC3605121

[59] Allen LJ, Burgin AM (2000) Comparison of deterministic and stochastic sis and sir models in discrete time. Math Biosci 163: 1–33. 10652843 10.1016/s0025-5564(99)00047-4

[60] Allen LJS (2003) An introduction to stochastic processes with applications in biology. New Jersey: Pearson Education Inc.

[61] Nasell I (2002) Stochastic models of some epidemic infections. Mathematical Biosciences 179: 1–19. 12047919 10.1016/s0025-5564(02)00098-6

[62] Vynnycky E, Fine PE (2000) Lifetime risks, incubation period, and serial interval of tuberculosis. Am J Epidemiol 152: 247–263. 10933272 10.1093/aje/152.3.247

[63] Dye C, Glaziou P, Floyd K, Raviglione M (2013) Prospects for tuberculosis elimination. Annu Rev Public Health 34: 271–286. 23244049 10.1146/annurev-publhealth-031912-114431

[64] Sanchez MA, Blower SM (1997) Uncertainty and sensitivity analysis of the basic reproductive rate. tuberculosis as an example. Am J Epidemiol 145: 1127–1137. 9199543 10.1093/oxfordjournals.aje.a009076

[65] Marino S, Hogue IB, Ray CJ, Kirschner DE (2008) A methodology for performing global uncertainty and sensitivity analysis in systems biology. J Theor Biol 254: 178–196. 18572196 10.1016/j.jtbi.2008.04.011 PMC2570191

[66] Hoare A, Regan DG, Wilson DP (2008) Sampling and sensitivity analyses tools (sasat) for computational modelling. Theor Biol Med Model 5: 4. 18304361 10.1186/1742-4682-5-4 PMC2292159

[67] Handel A, Longini Jr IM, Antia R (2009) Intervention strategies for an influenza pandemic taking into account secondary bacterial infections. Epidemics 1: 185–195. 20161493 10.1016/j.epidem.2009.09.001 PMC2796779

[68] Handel A, Longini IM, Antia R (2009) Antiviral resistance and the control of pandemic influenza: the roles of stochasticity, evolution and model details. Journal of theoretical biology 256: 117–125. 18952105 10.1016/j.jtbi.2008.09.021 PMC2624577

[69] Johnson P (2013) adaptivetau: Tau-leaping stochastic simulation. URL http://CRAN.R-project.org/package=adaptivetau. R package version 1.1.

[70] Cao Y, Gillespie DT, Petzold LR (2007) Adaptive explicit-implicit tau-leaping method with automatic tau selection. J Chem Phys 126: 224101. 17581038 10.1063/1.2745299

[71] Keeling MJ, Grenfell BT (1997) Disease extinction and community size: modeling the persistence of measles. Science 275: 65–67. 8974392 10.1126/science.275.5296.65

[72] Keeling MJ, Grenfell BT (2002) Understanding the persistence of measles: reconciling theory, simulation and observation. Proc Biol Sci 269: 335–343. 11886620 10.1098/rspb.2001.1898 PMC1690899

[73] Blower SM, McLean AR, Porco TC, Small PM, Hopewell PC, et al (1995) The intrinsic transmission dynamics of tuberculosis epidemics. Nat Med 1: 815–21. 7585186 10.1038/nm0895-815

[74] Nicol MP, Wilkinson RJ (2008) The clinical consequences of strain diversity in mycobacterium tuberculosis. Trans R Soc Trop Med Hyg 102: 955–965. 18513773 10.1016/j.trstmh.2008.03.025

[75] de Jong BC, Hill PC, Aiken A, Awine T, Antonio M, et al (2008) Progression to active tuberculosis, but not transmission, varies by mycobacterium tuberculosis lineage in the gambia. J Infect Dis 198: 1037–1043. 18702608 10.1086/591504 PMC2597014

[76] Hurtado AM, Hill KR, Rosenblatt W, Bender J, Scharmen T (2003) Longitudinal study of tuberculosis outcomes among immunologically naive ach natives of paraguay. Am J Phys Anthropol 121: 134–150. 12740957 10.1002/ajpa.10228

[77] Selwyn PA, Hartel D, Lewis VA, Schoenbaum EE, Vermund SH, et al (1989) A prospective study of the risk of tuberculosis among intravenous drug users with human immunodeficiency virus infection. N Engl J Med 320: 545–550. 2915665 10.1056/NEJM198903023200901

[78] Antonucci G, Girardi E, Raviglione MC, Ippolito G (1995) Risk factors for tuberculosis in hiv-infected persons. a prospective cohort study. the gruppo italiano di studio tubercolosi e aids (gista). JAMA 274: 143–148. 7596002 10.1001/jama.274.2.143

[79] Whalen CC, Johnson JL, Okwera A, Hom DL, Huebner R, et al (1997) A trial of three regimens to prevent tuberculosis in ugandan adults infected with the human immunodeficiency virus. uganda-case western reserve university research collaboration. N Engl J Med 337: 801–808. 9295239 10.1056/NEJM199709183371201

[80] Ottenhoff THM, Kaufmann SHE (2012) Vaccines against tuberculosis: where are we and where do we need to go? PLoS Pathog 8: e1002607. 22589713 10.1371/journal.ppat.1002607 PMC3349743

[81] Tiemersma EW, van der Werf MJ, Borgdorff MW, Williams BG, Nagelkerke NJ (2011) Natural history of tuberculosis: duration and fatality of untreated pulmonary tuberculosis in hiv negative patients: a systematic review. PLoS One 6: e17601. 21483732 10.1371/journal.pone.0017601 PMC3070694

[82] Trunz BB, Fine P, Dye C (2006) Effect of bcg vaccination on childhood tuberculous meningitis and miliary tuberculosis worldwide: a meta-analysis and assessment of cost-effectiveness. Lancet 367: 1173–1180. 16616560 10.1016/S0140-6736(06)68507-3

[83] van Leth F, van der Werf MJ, Borgdorff MW (2008) Prevalence of tuberculous infection and incidence of tuberculosis: a re-assessment of the styblo rule. Bull World Health Organ 86: 20–26. 18235886 10.2471/BLT.06.037804 PMC2647347

[84] Dowdy DW, Davis JL, den Boon S, Walter ND, Katamba A, et al (2013) Population-level impact of same-day microscopy and xpert mtb/rif for tuberculosis diagnosis in africa. PLoS One 8: e70485. 23950942 10.1371/journal.pone.0070485 PMC3741313

