# Supplementary material for: Modeling the Potential Impact of Host Population Survival on the Evolution of M. tuberculosis Latency
Source: PLoS One. 2014 Aug 26;9(8):e105721. doi: 10.1371/journal.pone.0105721 (PMC4144956; doi:10.1371/journal.pone.0105721)
Supplement: Text S1 — Additional results and explanations. (PDF) [file pone.0105721.s001.pdf]

Modeling the potential impact of host population survival on the evolution of *M. tuberculosis* latency

Supplementary Material

Nibiao Zheng[1], Christopher C. Whalen[2], Andreas Handel[2,*]

1 Institute of Bioinformatics, University of Georgia, Athens, GA 30602, USA
2 Department of Epidemiology and Biostatistics, College of Public Health, University of Georgia, Athens, GA 30602, USA

$*$ **E-mail: ahandel@uga.edu**

1 The impact of changes in individual parameters on MTB persistence and optimal activation

In the main text, we perform an uncertainty analysis and show, using a sampling approach, how changes in parameter values affect persistence and activation. Here, we focus on varying each parameter individually and investigate how this affects the results. We vary each model parameter, one at a time, and for each value of the model parameter compute persistence as a function of activation rate. Figure 1 shows heat map plots of persistence, P, as a function of each parameter and activation rate.

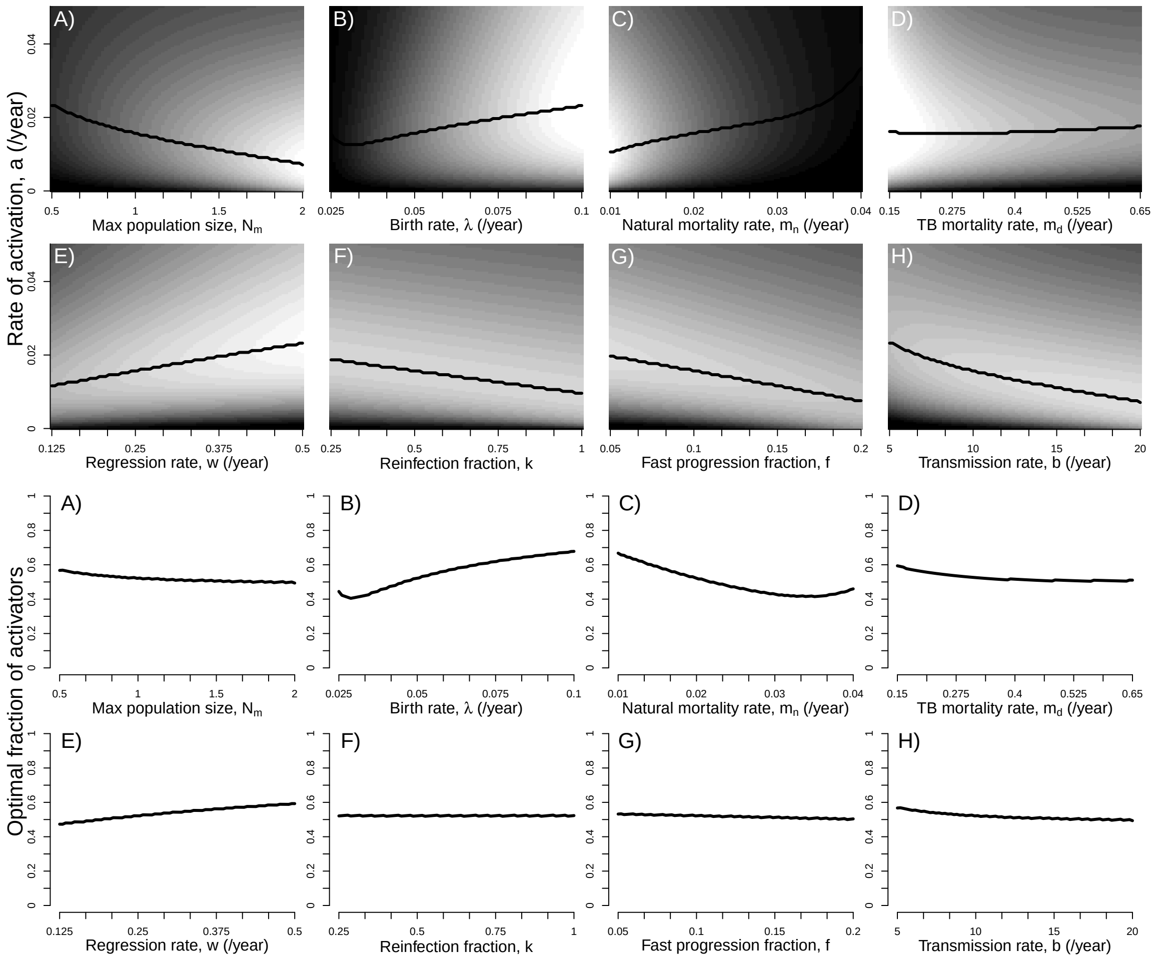

Figure 1. Top: Heat maps of persistence, P, as a function of activation rate and each model parameter. Remaining parameter values are as given in the table in the main text. Lighter colors represent higher values of persistence. The black line shows the rate of activation that optimizes persistence. Bottom: Optimal fraction of activators as each parameter is changed.

Not surprisingly, an increased maximum population size, N_m and increased birth rate λ, lead to better persistence, while increased natural or disease-induced mortality, m_n and m_d lead to reduced persistence.

An increase in regression rate, the rate at which infectious, diseased hosts can revert to the asymptomatic and non-infectious latent stage, leads to some increase in persistence. Since regression acts as

the reverse process of TB activation, an increase in regression rate leads to a concomitant increase in the optimal rate of activation. If it were possible to increase regression rates (e.g. through more and better treatment) and reduce the rate of activation (e.g. through a novel vaccine), one could drive MTB toward the lower right corner of the figure, which shows very low persistence, i.e. a high chance of extinction.

For activation after reinfection, one finds that overall persistence changes minimally. Since reinfection leads to more hosts with TB disease, an increase in this quantity is balanced by a decrease for the optimal activation rate.

For fast progression, the same balance between increased fast progression and reduced optimal activation holds. Persistence along the optimal activation rate curve slightly decreases as fast progressors increase. This suggests that fast progression is not beneficial for MTB persistence. Instead, having all hosts move through a latent stage is best. The fact that a small fraction of hosts develop disease rapidly is presumably due to host heterogeneity, and not an evolutionary strategy of MTB.

An increase in transmission rate leads to a modest increase in persistence. The optimal activation decreases slightly, because a higher transmissibility requires few hosts with infectious TB disease at any given time.

2 The impact of changes in individual parameter for the non-steady state scenario

Figure 2 shows heatmap plots for individual parameter changes, now plotting the non-steady state persistence measure P_m. The results are almost identical to the steady state scenario. Careful inspection of the steady-state and non-steady state figures shows minor differences for some parameter values, but those are so minor as to be virtually indistinguishable. This is – as explained in the main text – due to the fact that TB has a relatively “slow” disease dynamics [1], without pronounced outbreak peaks and minima. Therefore, the disease reaches the steady state without a large contraction after the first outbreak, leading to essentially the same results as for the steady state.

3 Persistence for a non-exponential lifespan model

In the main text, we studied a standard compartmental ordinary differential model. For models formulated in such a way, the implicit assumption is that the distribution of times spent in any compartment is exponentially distributed. This is a common, convenient, but somewhat unrealistic assumption. It has been previously shown and discussed that while the exponentially distributed assumptions generally lead to similar results with regard to steady states, they do at times alter the outcomes [2–4].

One can modify the model in several ways to introduce more realistic distributions of lifespans [5]. One simple and flexible way is through the use of additional (often referred to as dummy) compartments citelloyd01,keeling07. By splitting each compartment into multiple compartments, the transition out of a given biological compartment (i.e. S, L, I in our model) becomes gamma-distributed, resembling more of the mortality pattern observed for humans. We implement such a modification for our model. The

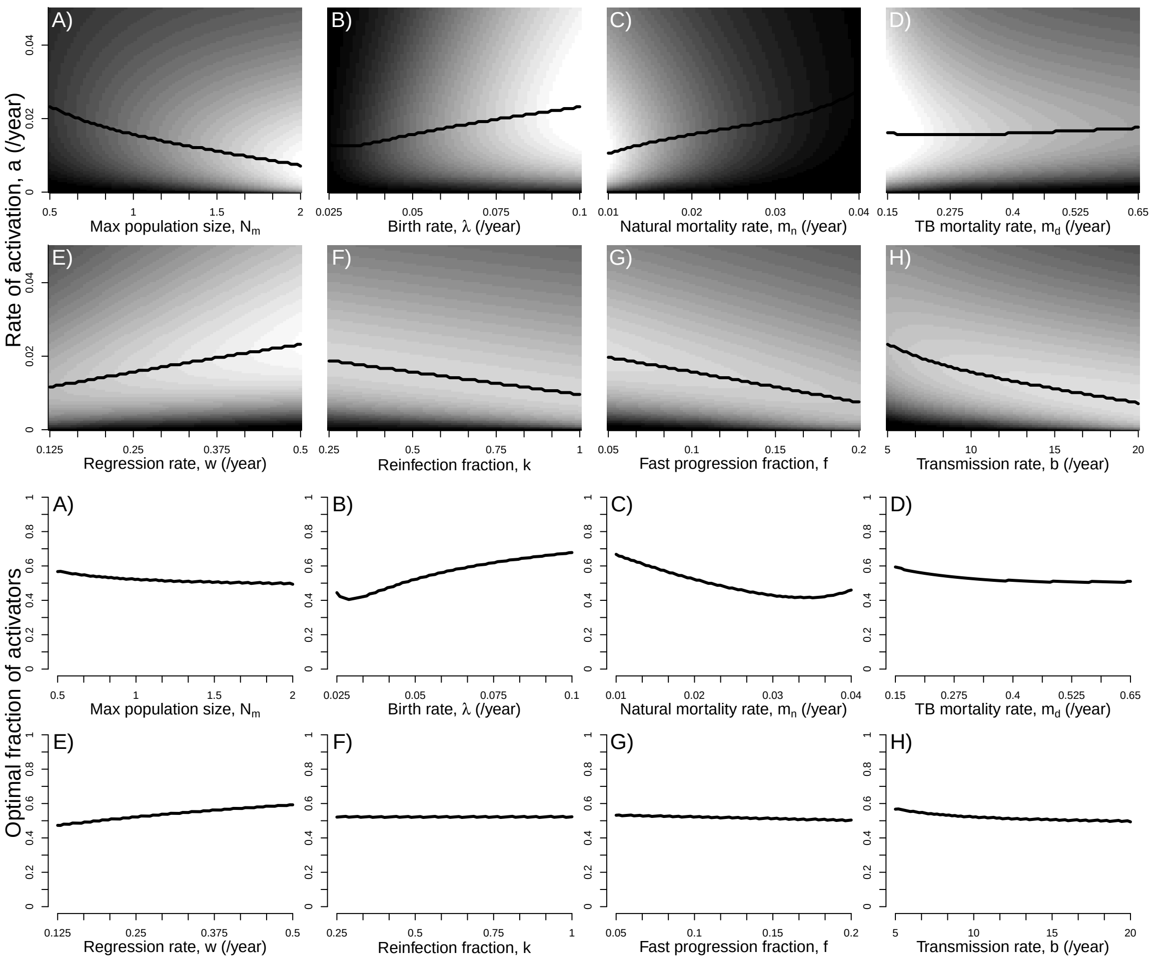

Figure 2. Top: Persistence, P_m, as a function of activation rate and each model parameter. Remaining parameter values are as given in the table in the main text. Lighter colors represent higher values of persistence. The black line shows the rate of activation that optimizes persistence. Bottom: Optimal fraction of activators as each parameter is changed.

modified equations are given by

$$\frac{dS_1}{dt} = \lambda N(1-\frac{N}{N_m}) - bI_tS_1 - \delta S_1 \quad (1)$$

$$\frac{dS_j}{dt} = \delta S_{j-1} - bI_tS_j - \delta S_j, \qquad j = 2...J-1 \quad (2)$$

$$\frac{dS_J}{dt} = \delta S_{J-1} - bI_tS_J - \delta S_J \quad (3)$$

$$\frac{dL_1}{dt} = (1-f)bI_tS_1 + wI_1 - aL_1 - kfbI_tL_1 - \delta L_1 \quad (4)$$

$$\frac{dL_j}{dt} = (1-f)bI_tS_j + wI_j - aL_j - kfbI_tL_j + \delta L_{j-1} - \delta L_j, \qquad j = 2...J-1 \quad (5)$$

$$\frac{dL_J}{dt} = (1-f)bI_tS_J + wI_J - aL_J - kfbI_tL_J + \delta L_{J-1} - \delta L_J \quad (6)$$

$$\frac{dI_1}{dt} = fbI_tS_1 + aL_1 + kfbI_tL_1 - (w+m_d)I_1 - \delta I_1 \quad (7)$$

$$\frac{dI_j}{dt} = fbI_tS_j + aL_j + kfbI_tL_j - (w+m_d)I_j + \delta I_{j-1} - \delta I_j, \qquad j = 2...J-1 \quad (8)$$

$$\frac{dI_J}{dt} = fbI_tS_J + aL_J + kfbI_tL_J - (w+m_d)I_J - \delta I_J \quad (9)$$

where we defined $S_t = \sum_{j=1}^{J} S_j$, $L_t = \sum_{j=1}^{J} L_j$ and $I_t = \sum_{j=1}^{J} I_j$. By setting $\delta = Jm_n$, the average lifespan of hosts is again given by $1/m_n$. The number of compartments, J, dictates the distribution of the transition, with the cases $J = 1$ corresponding to the exponential distribution of the simple ODE model and $J \to \infty$ turning into delta-distributed transitions [2].

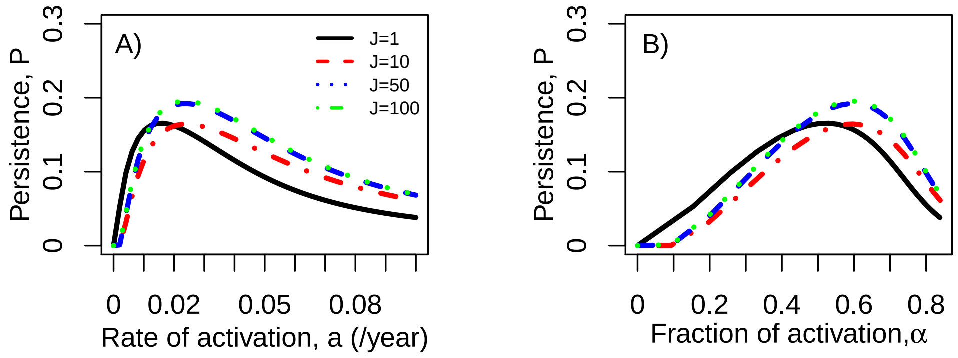

Figure 3. Persistence, P, as a function of a) activation rate and b) fraction of activation. Results are shown for $J = 1$ (the ODE model from the main text), and $J = 10, 50, 100$ dummy compartments. All parameters are as described for the equivalent figure in the main text.

As figure 3 shows, the change of the life-span distribution from an exponential to a gamma distributed leads to a small shift in the persistence curve. The overall shape and intermediate optimum remain. This echoes findings from previous studies of TB models with non-exponential distributions that did not find strong differences for other outcomes of study [6, 7].

4 Persistence in the presence of population growth or decline

In the main text, we considered a model with births and mortality balancing each other at the disease-free steady state and therefore no population growth. Here, we investigate how results would change in the presence of either growing or declining populations. To that end, we initialize our stochastic simulation at the model's steady state. We then either increase or decrease the birth rate λ by 20%. This leads to an underlying growth or decline in the population. As expected, a growing population leads to overall better persistence, while a declining population reduces the probability of extinction (figure 4). The rate of optimal activation also shifts somewhat, while the overall shape and the finding that an intermediate rate is optimal do not change.

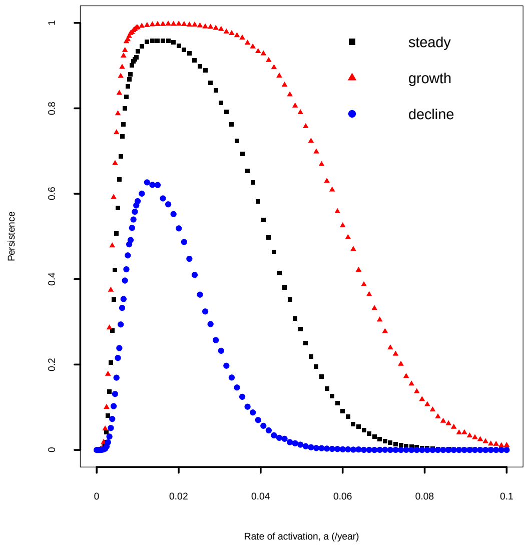

Figure 4. Persistence probability obtained from the stochastic model as a function of activation rate. Results are shown for a fixed (black squares), growing (red triangles) and declining (blue circles) population. Starting at steady state, we ran the model for 1000 years and counted the numbers of runs for which any latent or infectious hosts were still present at the end of the simulation. The model was run 1000 times, and a maximum population of size $N_m = 100$ was used. All parameter values are as reported in the table in the main text. For the growing or declining populations, we either increased or decreased the birth rate λ by 20% at the beginning of each stochastic run.

References

1. Blower SM, McLean AR, Porco TC, Small PM, Hopewell PC, et al. (1995) The intrinsic transmission dynamics of tuberculosis epidemics. Nat Med 1: 815-21.

2. Lloyd AL (2001) The dependence of viral parameter estimates on the assumed viral life cycle: limitations of studies of viral load data. Proc Biol Sci 268: 847–854.

3. Wearing HJ, Rohani P, Keeling MJ (2005) Appropriate models for the management of infectious diseases. PLoS Med 2: e174.

4. Keeling M, Rohani P (2007) Modeling Infectious Diseases in Humans and Animals. Princeton University Press.

5. Holder BP, Beauchemin CAA (2011) Exploring the effect of biological delays in kinetic models of influenza within a host or cell culture. BMC Public Health 11 Suppl 1: S10.

6. Feng Z, Huang W, Castillo-Chavez C (2001) On the role of variable latent periods in mathematical models for tuberculosis. Journal of Dynamics and Differential Equations 13: 425–452.

7. Castillo-Chavez C, Song B (2004) Dynamical models of tuberculosis and their applications. Math Biosci Eng 1: 361–404.
